# Outbreak report: a nosocomial outbreak of vancomycin resistant *enterococci* in a solid organ transplant unit

**DOI:** 10.1186/s13756-018-0374-5

**Published:** 2018-07-18

**Authors:** Peter Kreidl, Astrid Mayr, Guido Hinterberger, Michael Berktold, Ludwig Knabl, Stefan Fuchs, Wilfried Posch, Stephan Eschertzhuber, Alois Obwegeser, Cornelia Lass-Flörl, Dorothea Orth-Höller

**Affiliations:** 1 Department of Hygiene, Microbiology and Social Medicine, Division of Hygiene and Medical Microbiology, Medical University of Innsbruck, Innsbruck, Schoepfstr. 41, 6020 Innsbruck, Austria; 2 Department of Anesthesia and Critical Care, Centre of Operative Medicine, Medical University of Innsbruck, Anichstr. 35, 6020 Innsbruck, Austria; 3 Department of Neurosurgery, University Hospital of Innsbruck, Anichstr. 35, 6020 Innsbruck, Austria

**Keywords:** Solid organ transplant unit, Vancomycin resistant *enterococci*, Outbreak, Survival benefit, Infection control

## Abstract

**Background:**

Vancomycin resistant *enterococci* (VRE) are an emerging problem in health care settings. The purpose of the investigation was to assess the extent of the outbreak including environmental contamination and to limit further transmission.

**Methods:**

We used retrospective patient and laboratory data including pulse field gel electrophoresis (PFGE) typing and virulence and resistance gene analysis. For comparison of medians the Mann-Whitney and for comparison of proportions the Fisher exact tests were used.

**Results:**

PFGE typing of VRE strains of an outbreak of 15 VRE cases in a solid transplant unit revealed that nine of the cases belonged to one identical pattern (A), which was only found twice in the environment. Eleven further positive environmental samples showed a different, but identical PFGE pattern E. Only one patient was infected with this environmental strain.

Two of nine (22.2%) PFGE A, but nine of eleven (81.2%) PFGE E samples were positive for gelatinase E (*p* = 0.01), which is described as enhancing biofilm production, suggesting a survival benefit for this strain on inanimate surfaces.

**Conclusion:**

Routine disinfection was not able to stop the cluster, but after repeated enforcement of the infection prevention and control (IPC) bundle such as training, strict adherence to hand hygiene and surface disinfection no further cases were observed. We conclude that certain VRE strains predominate in the environment whereas others predominate in humans. Enforcement of the IPC bundle is essential for controlling VRE outbreaks and reducing further transmission.

## Background

Vancomycin resistant *enterococci* (VRE) are important causes of morbidity and mortality especially in health care settings where they easily disseminate [1]. Effective treatment is limited and thus VRE remain a major challenge for infection control.

Vancomycin resistance of invasive *Enterococcus faecium* isolates ranges between 0 and 46.3% in countries of the European Union and European Economic Area (EU/EEA). The proportion of VRE in Austria (4.3% in 2016) was below the EU population weighted mean of 11.8% (95% confidence interval (CI) 11–13%) [2]. Monoclonal and polyclonal outbreaks of VRE were described worldwide; the largest reported outbreaks comprised up to 72 cases [3].

The most important transmission-routes of VRE are still not fully understood, but cross contamination via the hands of health care workers’ (HCW) is believed to play a major role. HCW act as a vector between colonized or infected patients, inanimate surfaces and previously unaffected patients [4]. The reservoir for VRE is believed to be the human intestine [5].

Known risk factors for acquisition of VRE include previous use of antibiotics, prolonged hospital stay, underlying diseases, admission to high risk departments such as oncology, hematology, transplant or intensive care units (ICU), the nurse to patient ratio, and occupancy to a room where previously a patient harboring VRE was admitted [6]. The transmission in high risk wards via contaminated inanimate surfaces is believed to be important but it remains a challenge to quantify the attributable risk [6] of environmental contamination.

We describe an outbreak which was identified on 7th January 2017 after detection of five cases within 2 months following the Orion statement [7].

The objective of this investigation was to assess the extent of the outbreak including the environmental contamination, to limit further transmission and to evaluate the effectiveness of implemented control measures. Further important objectives were to foster infection prevention and control (IPC) education by the infection control team (ICT) of the University Hospital Innsbruck (LKI).

## Methods

### Study design

We used retrospective data analysis including PFGE typing, analysis of virulence and resistance genes and patient relevant data from the hospital records.

### Participants

Participants were patients admitted to the solid organ transplant unit between 15th November 2016 and 30th June 2017 with laboratory confirmation of VRE. Colonization was defined as laboratory confirmation of VRE in a sample of a patient from a non-sterile site without signs of infection such as fever > = 38.5 °C. Infection was defined as laboratory confirmation of VRE in a sample of a patient from a normally sterile site or signs of infection such as fever > = 38.5°.

### Case definition

We defined a VRE case as a patient admitted to the transplant unit during the observation period with laboratory confirmation of VRE in at least one sample irrespective of the location or type of sampling. We defined a cluster case as a VRE case with the outbreak PFGE pattern A identified between December 2017 and June 2018 and admitted to the transplant unit.

### Setting

The period of the outbreak lasted from 15 November 2016 until 30 June 2017.

The LKI is a 1600-bed tertiary-care hospital with several departments including a solid organ transplant unit.

The setting of the outbreak was the ward of the transplant unit consisting of 15 beds in seven rooms (one to four beds per room) and an adjacent intensive care unit (ICU) consisting of eight beds situated in three separate rooms. Patients were transferred between the standard care unit and the ICU depending on their medical condition. The ICT consisted of one senior hospital hygiene specialist and a local hygiene team.

### Routine IPC protocol

The internal IPC protocol included the following recommendations: 1) twice daily unsupervised routine disinfection of inanimate surfaces of the patient close environment with an aldehyde-free-broad-spectrum disinfectant containing quaternary ammonium compounds (QACs, 2% TPH protect, Schülke & Mayr GmbH, Vienna, Austria) including once daily patient charts, 2) terminal cleaning and disinfection of patient rooms with the above mentioned disinfectant and 3) hand hygiene (HH) based on the WHO approach “My Five Moments for Hand Hygiene” [8] using alcohol-based detergents (Sterillium®, BODE Chemie GmbH, Hamburg, Germany). Surveillance of hand hygiene compliance is conducted through bi-annual audits based on the protocol from the Robert Koch Institute [9], measuring adherence to HH. The outcomes of the audits were categorized as sufficient or insufficient per unit but lacked a coding system to quantify the adherence to HH on an individual basis. Annual trainings of the IPC protocol through verbal presentations and ward round sessions were routinely conducted. Data on baseline IPC knowledge of HCW were not routinely collected. IPC guidelines did not require pre-admission VRE screening of patients, unless 1) patients had a positive history of exposure in a high endemic country, 2) were transferred from a long-term-care-facility or 3) were previously known having been infected or colonized with VRE.

According to the IPC protocol, each newly identified patient either colonized or infected by VRE irrespective of the patient being part of an outbreak, triggered a routine infection control response consisting of following measures: isolation precautions, defined as placement in a single room, if possible, and, contact precautions depending on the estimated potential for transmission including personal protective equipment.

### Intervention during the outbreak

During the outbreak the following additional measures were implemented: Re-emphasizing strict adherence to HH and to environmental disinfection including unannounced microbiological monitoring of the environment and HCW’s hands, repeated sampling of stool or rectal swabs of the VRE positive patients, newly admitted patients and rectal screening of HCWs. Rectal swabbing of patients was implemented as precautionary measure to limit the extent of the outbreak. Self-administered rectal swabbing of HCWs was conducted on voluntary basis upon the urgency of the situation. Thus informed consent was not obtained.

Frequency of ward round training was increased. The choice of HH antisepsis and environmental disinfection during the outbreak remained the same and compliance with HH was observed by the ICT. No additional decontamination procedures such as fogging or steaming were implemented.

### Culturing and typing

#### Patient samples

We collected routine clinical samples and rectal screening swabs from patients according to the IPC protocol (no informed consent was requested for rectal sampling), cultivated them on blood agar (Becton Dickinson, Heidelberg, Germany) and selective ChromID VRE Agar (Biomerieux, Marcy-l’Étoile, France) and incubated them for 48 h at 37 °C under aerobic conditions. Suspect colonies were identified by matrix-assisted laser desorption ionization time-of-flight mass spectrometry (MALDI-TOF, Bruker, Bremen, Germany). Antibiotic susceptibility testing was performed according to the European Committee on Antimicrobial Susceptibility Testing (EUCAST version 6.0, 2016) protocol [10]. *Enterococci* were classified as VRE if the minimal inhibitory concentration of vancomycin was above 4 mg/L identified by E-Test (Biomerieux, Marcy-l’Étoile, France) [11]. Additional patient data such as diagnosis, duration of hospital stay, outcome, type and time of transplantation were obtained from the medical records of the LKI. 

#### Environmental samples

Unheralded sampling of environment was conducted according to Galvins protocol (Galvin) using Columbia-III-Sheep blood agar 5% (Oxoid Limited, Basingstoke, UK) or Tryptic soy agar with neutralizers (VWR International, Radnor, USA) contact plates with a press on time of 10 seconds without any lateral movement. Surfaces were selected at different locations close and distant from the respective patients [12] during three occasions: immediately after the alert of the cluster on 18 January, and twice after enforcement of control measures (30th January and 2nd February 2017, respectively). Data recorded were date, type and location of sampling, laboratory findings and PFGE results. Direct unheralded observation of hygienic measures was conducted to identify potential gaps of adherence to infection control measures. Patient close samples were defined as samples obtained from the vicinity of patients such as the bedrail, bedframe, beside tables, clamps for urine-bags, drug perfusors, stethoscope and bedside monitors [13].

Microbial monitoring of HCWs hands was performed by imprinting all fingertips including the thumb onto Columbia agar containing 5% sheep blood (Becton, Dickenson and Company, Franklin Lakes, USA) for approximately 5 seconds [14, 15]. Confirmation of VRE was conducted as described above.

#### PFGE

Molecular Typing was performed using pulse field gel electrophoresis (PFGE) according to the protocols published by the Centers for Disease Control and Prevention [16]. Extracted bacterial DNA was restricted using Smal enzyme. Strain typing was performed by manual PFGE restriction pattern analysis [17] and by computational analysis of band differences using GelJ Software Version 2 [18]. Dendrogram analysis of PFGE patterns was performed using Dice algorithm and Single Linkage clustering. Genotypically related strains with similarity > 90% were considered belonging to the same pattern.

#### Genetic analysis

Vancomycin resistance genotypes were determined according to the protocol from Jayaratne et al. [19]. The enterococcal surface antigen was identified according to the protocol by Toledo-Arana [20], the cytolysin activator according to Vankerckhoven [21] and the gelatinase E according to Hancock’s protocol [22].

### Infection related outcomes

Infection related outcomes, including colonization, infection and patient survival were assessed.

The specimen processing and turn-around time were performed according to local laboratory protocol from 2016.

### Sample size

The sample size was limited to the number of identified patients (*n* = 15).

### Statistical methods

Variables of interest were stored in excel database including age, gender, number of isolates obtained including positives, date of available laboratory result, source and material of samples, length of hospital stay, previous antibiotic treatment, previous confirmation of VRE in any sample, diagnosis, other identified pathogens, and patient outcome.

Descriptive analysis was performed using Epi info version 7.2.2.2. (CDC Atlanta). For comparison of medians the Mann-Whitney and for comparison of proportions the Fisher exact tests were used. A *p*-value of < 0.05 was considered statistically significant.

## Results

### Event description

On 7th January 2017 the outbreak response team of the LKI informed the transplant unit about a potential outbreak of VRE.

Analysis of historical data revealed that between May 2012 and October 2016, a total of 53 VRE cases were identified at this unit. The median incidence was one (0–3) case per month and the annual median incidence was 11 cases (6–16 cases) per year for the period 2013–2016. In comparison to previous years, the observed number of five VRE cases in a period of less than 2 months clearly exceeded the expected value suggesting an outbreak.

### VRE cases

After a period of 4 months without any detection of VRE from the transplant unit, the potential index case was confirmed on 15th November 2016. He was a 58 year old male suffering from IgA Nephritis who received a kidney transplant on 12th November 2016, 3 days prior to the diagnosis of VRE in a single sample of a retroperitoneal surgical-site-drainage. The patient was classified as being infected.

**Fig. 1:**
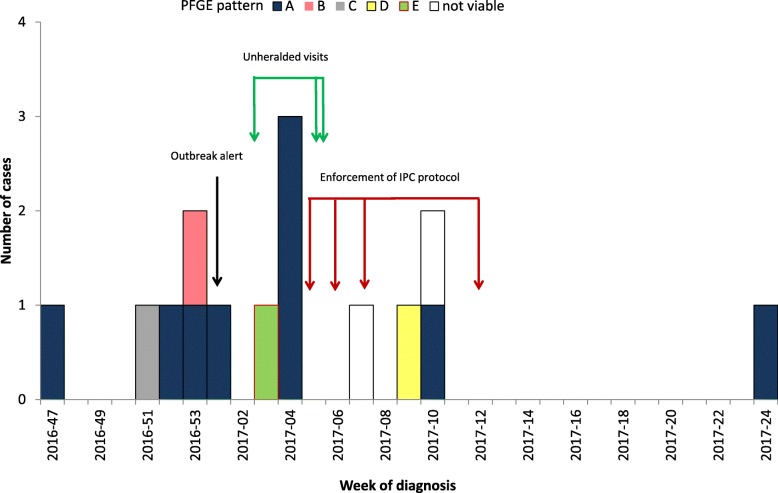
Number of cases by PFGE pattern, week of diagnosis and main interventions (*n* = 15)

Between 15th November 2016 and 30th June 2017, VRE was confirmed in at least one sample of 14 additional patients (Fig. 1). Of these, ten patients (66.7%) were males with a median age of 56.5 years (50–71 years). The five female patients showed a median age of 70 years (60–79 years) (*p* = 0.003) (Table 1).

**Table 1:** Patient characteristics

	Categories	N/total or median (mean; range)	%
Demographic characteristics	Males	5/15	67
	Age	59 yrs. (61.3 yrs.; 50–79 yrs)	–
Clinical findings	Infected (versus colonized)	12/15	80
	Deceased	3/15	20
Laboratory sampling	Number of samples investigated per patient	24 (31; 8–105)	–
	Number of positive samples per patient	2 (6.1; 1–52)	–
Laboratory findings	PFGE pattern A (cluster strain)	9/13	69
	PFGE pattern E (environmental strain)	1/13	8
	other PFGE patterns	3/13 (one each)	23

VRE was first isolated twice from blood cultures, five times from urine samples, four times from wound drainage and once each from a tissue, stool, and rectal swab and from a broncho-alveolar lavage sample. Three of the patients were classified as being colonized, the remaining twelve as being infected with VRE. The median length of hospital stay was 24 days (10–112 days). All but one patient underwent transplantation and received immunosuppression. Twelve patients survived (80%) and three died (Table 1). In all patients *Enterococcus faecium* was identified, one patient harbored a tigecycline resistant, two a linezolid resistant and fourteen teicoplanin resistant strains.

### PFGE patterns of cases

**Fig. 2:**
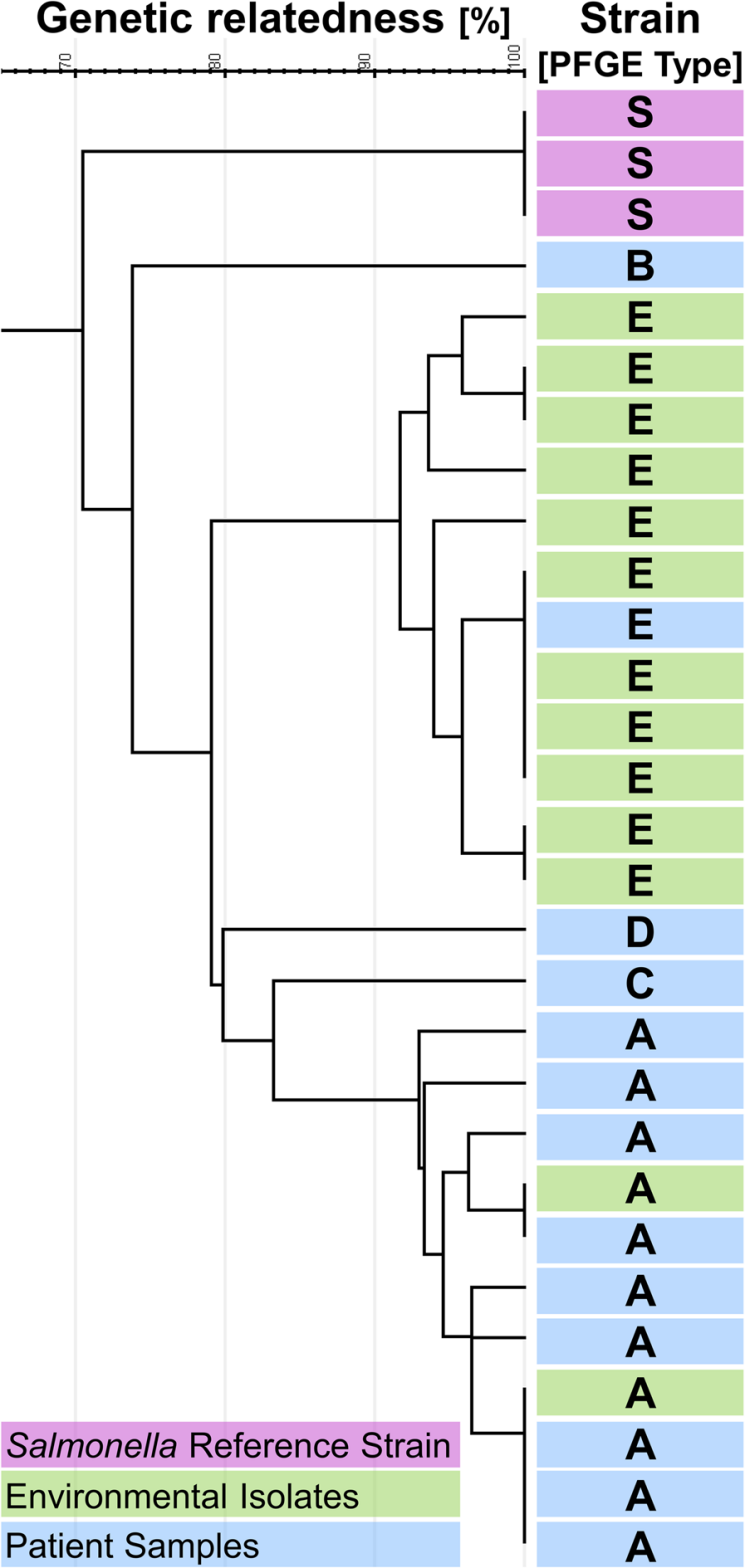
Dendrogram of PFGE patterns of human (*n* = 13; pattern A = 9, one each pattern B, C, D, E and environmental VRE strains (*n* = 13; pattern E = 11, pattern A = 2) including 3 control strains

PFGE patterns were available for 13 of 15 patients (86.7%). Among those, five different PFGE patterns were identified and subsequently classified as patterns A to E. Nine of the thirteen cases (69.2%) were classified as PFGE pattern A (further called cluster strain, Table 1, Fig. 2).

All 10 of the 15 patient isolates (66.7%) available for vancomycin resistance genotyping (*van*R) revealed *van*A, irrespective of their PFGE pattern. The only patient isolate with pattern E, was not viable.

### Cluster cases

In total, 194 patient samples were obtained from cluster cases, in 26 of them (13.4%) VRE was confirmed. The median number of samples investigated per cluster case was 20 (12–34) and the median number of positive samples was one (1–12). Five patients only had one sample positive for VRE. The median duration of confirmation between the first and the last positive sample was 4 days (1–33 days). The median interval between admission and first isolation of VRE was 13 days (same day-22 days) (*n* = 14). One additional patient already tested positive in another unit during the outbreak period, but the isolate was different from the cluster strain (PFGE C). From all other cases no information of previous VRE carriage was available as no routine VRE screening was conducted prior to this outbreak. VRE was first identified in following materials from the nine cluster cases: Blood (*n* = 1), (wound) drains (*n* = 4), rectal swab (*n* = 1), and urine (*n* = 3).

### VRE screening

Pre-admission rectal screening was implemented after the outbreak alert. Among 169 patients screened until the end of 2017, only one was found to be VRE positive (0.6%).

### Environmental and hand samples

**Fig. 3:**
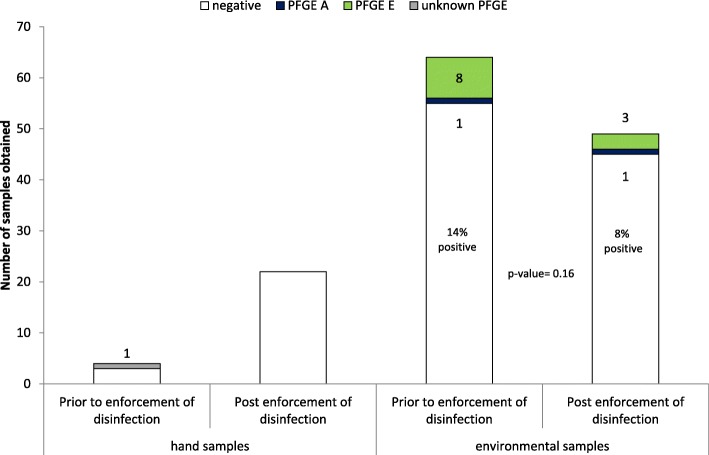
Number and results of hand and environmental samples by intervention (*n* = 139)

A total of 139 hand (*n* = 26; 18.7%) and environmental (*n* = 113, 81.3%) samples were obtained during three unheralded on-site visits; nearly half of them (*n* = 68, 48.9%) were obtained prior to the first enforcement of control measures. More samples taken prior to enhanced disinfection (*n* = 10; 14.7%) were positive for VRE compared to post disinfection sampling (*n* = 4; 5.6%) (*p* = 0.07). Among the thirteen VRE positive environmental samples the cluster strain (PFGE A) was identified twice: once from a clamp of a urine-bag prior and once on a patients chart after the first enforcement of control measures. All other eleven VRE positive environmental samples revealed one identical PFGE pattern E which was distinct from the patient cluster strain (Fig. 2). Pattern E was confirmed only in one patient, who was admitted 2 days prior to the first environmental sampling. Eight of these positive non-cluster strains (PFGE E) were identified during the first on-site visit. Six of them were determined in the patient close environment and two outside patient rooms. During the third visit the environmental PFGE E strain was identified again three times. Once from the patient close environment (urine clamp bag) of the patient infected with PFGE E; and twice from a bed located in the corridor (Fig. 3). All tested environmental strains were *van*A positive.

One VRE positive hand sample of a HCW was not available for PFGE typing.

### Virulence genes

Twenty three samples were available for virulence gene testing (13 environmental and 10 patient samples).

Two of nine (22.2%) PFGE A (six patient samples), but nine of 11 (81.2%) PFGE E (all environmental samples) were positive for gelatinase E (*p* = 0.0123). The two gelatinase E positive PFGE A samples were one human sample from a patient already discharged at the time of the environmental sampling and one environmental sample from a urinary bag clamp obtained during the first on-site visit which could not be linked to a specific patient.

All investigated samples were positive for enterococcal surface protein and negative for cytolysin activator.

### Control measures

The first of the three unheralded on-site visit including environmental and hand sampling was undertaken on 18th January 2017. Lack of adherence to hand hygiene was observed and therefore enforcement of control measures was stressed by the infection control hygiene team. At the time of this first visit, five patients were admitted (two with PFGE A, one each with PFGE B and E and one without a viable isolate) and six already discharged (five of whom with PFGE A and one with PFGE C).

The second sampling on 30th January, performed after extended cleaning and disinfection procedures towards patients’ distant zones did not detect any environmental contamination. Rectal screening targeting all staff was conducted the same day. The participation rate was 79% (94 of 119), all samples remained negative. Non-participation was mainly due to absence; No consent was obtained as participation was voluntary. In the current IPC protocol HCW hand sampling but no rectal swabbing is included.

During the last on-site visit on 2nd February, VRE was detected in four of 47 (8.51%) environmental samples. Three of those revealed PFGE E, all from patient-close sites of the patient infected with PFGE E, the cluster strain (PFGE A) was isolated from a patient chart. At the time of the third visit, five patients (two with PFGE A) were still admitted to the ward; four further cases, two with the cluster PFGE A were identified later. Enforcement of IPC measures was rigorously stressed again and four ward round sessions were conducted. Insufficient adherence to HH according to the IPC protocol was documented during the first visit. During the second and third visit the adherence to HH was evaluated to be sufficient. The terminal cleaning was audited by the ICT and adherence was defined as sufficient already at the first visit. The effect of teaching was not measured.

## Discussion

We describe an outbreak of fifteen VRE cases in a solid organ transplant unit in late 2016 and early 2017, of which nine patients revealed an identical PFGE pattern A, further called the cluster strain.

During the first visit six VRE positive environmental samples were identified in the patient close environment which suggests lack of effective decontamination despite the audited terminal disinfection which was considered sufficient. Additionally, lack of adherence to strict hand hygiene was observed and compliance to HH was classified as insufficient. The two positive samples identified from a laundry rack suggest cross-contamination via HCW hands, as all patients were immobile. The fact, that the majority of environmental strains were identical - although distinct from the outbreak strain - may suggest that also prior to the first sampling environmental contamination via HCW hands may have occurred. HH adherence during audits improved from insufficient during the first visit to sufficient during the second and third visit.

Enforcement of ICP measures resulted in a decrease of the proportion of VRE contaminated environmental samples, although it was not significant (Fig. 3). Nevertheless, reconfirmation of the non-cluster strain during the third site visit may be due to recontamination and insufficient compliance to hand hygiene. In addition, the confirmation of the cluster strain on a patient chart after enforcement of control measures suggests the cross-contamination via HCW hands as well as ineffective decontamination of the patient chart. Decontamination of patient charts is described as effective measure to decrease horizontal transfer of organisms and to prevent transmission of health care associated infections [23, 24].

We speculate that the lack of strict adherence to adequate hand hygiene might have led to contamination of the environment. This is supported by the facts that firstly, contamination was observed in patient distant areas such as laundry racks and secondly, that contaminated patient charts are described being a result of lack of adherence to recommended HH [25]. This may have played an important role in triggering the outbreak. Tight working spaces in the ICU unit favor the patient-to-patient contact [26] and may have resulted in a higher risk for transmission of VRE.

Hayden concludes that the role of environmental contamination in nosocomial cross-transmission of VRE is still unresolved but enforcement of environmental decontamination was both associated with reduction of surface contamination and contamination of HCWs’ hands despite only moderate adherence to proper hand hygiene [27]. Proper environmental decontamination may reduce the risk of VRE outbreaks in hospital settings as suggested in previous studies. Dancer et al. [28] suggest to focus more on patient close hand-touch sites rather than on general surfaces and bathrooms.

The observed higher median age of male VRE patients rather reflects the distribution of admitted patients than being associated with VRE colonization or infection [29].

The pre-admission rectal screening implemented since the identification of the outbreak revealed less than one positive per hundred investigated patients. VRE screening in a tertiary hospital setting is suggested to decrease the incidence in routine patient care and even more important in an outbreak situation [30–32]. Therefore we speculate that most of the cluster cases acquired the VRE during the hospital stay.

The cluster strain PFGE A was identified only twice in the environment; all other environmental strains revealed an identical PFGE E pattern. This PFGE E pattern was found only in one patient. The fact, that significantly more PFGE E compared to PFGE A strains were producing gelatinase E, which is described as enhancing biofilm production, suggests a survival benefit on inanimate surfaces [33]. Accumulation of mobile genetic elements including plasmids, pathogenicity islands, resistance transposons other fitness islands, phages and surface types [34, 35] may also have contributed to better survival in the environment.

The limitations of our study are that we used retrospective data analysis which made selection of appropriate controls difficult. Also logistical reasons hindered us to conduct a case control study. Therefore we were neither able to identify risk factors for acquisition of VRE nor to identify the source of infection. Furthermore, neither retrospective data where patients were located within the ward at the time of admission nor information on staffing was available. A higher patient-staff ratio might have influenced the transmission risk. The only PFGE E strain from the patient could not be recovered from the archived skim-milk stock and was hence also not available for genotyping.

## Conclusions

We conclude that enforcement of strict adherence to the existing IPC protocol and assessment of adherence are essential for controlling VRE outbreaks and reducing further transmission. We cannot exclude the role of other components in the multi-modal IPC plan instituted which ultimately may have played a role in the control of the outbreak. Unheralded visits are useful tools to further improve adherence to IPC protocols. Frequently touched surfaces such as patient charts are difficult to decontaminate and therefore require particular attention. We conclude that this outbreak has resulted in better control of future VRE outbreaks. HCWs reported a better understanding for the need of rigorous adherence to control measures. We expect further sporadic cases in future.

## Abbreviations

CI: Confidence interval
EU/EEA: European Union and European Economic area
HCW: Health care worker
HH: Hand hygiene
ICT: Infection control team
ICU: Intensive care unit
IPC: Infection prevention and control
LKI: University Hospital Innsbruck (Landeskrankenhaus Innsbruck)
PFGE: Pulse filed gel electrophoresis
VRE: Vancomycin resistant *enterococci*
